# Indications for conservative management of scoliosis (guidelines)

**DOI:** 10.1186/1748-7161-1-5

**Published:** 2006-05-08

**Authors:** SOSORT guideline committee, Hans-Rudolf Weiss, Stefano Negrini, Manuel Rigo, Tomasz Kotwicki, Martha C Hawes, Theodoros B Grivas, Toru Maruyama, Franz Landauer

**Affiliations:** 1 Asklepios Katharina Schroth Spinal Deformities Rehabilitation Centre, Bad Sobernheim, Germany; 2 ISICO (Italian Scientific Spine Institute), Milan, Italy; 3 Instituto Èlena Salvá, Barcelona, Spain; 4 University of Medical Sciences, Poznan, Poland; 5 University of Arizona, Tucson AZ 85721, USA; 6 Orthopaedic Department "Thriasion" General Hospital, Magula, Athens, Greece; 7 Department of Orthopaedic Surgery, Teikyo University School of Medicine, 2-11-1 Kaga, Itabashi-ku, Tokyo 173-8605, Japan; 8 Landesklinik für Orthopädie, Müllner Hauptstr. 48, A-5020 Salzburg, Austria

## Abstract

This guideline has been discussed by the SOSORT guideline committee prior to the SOSORT consensus meeting in Milan, January 2005 and published in its first version on the SOSORT homepage: . After the meeting it again has been discussed by the members of the SOSORT guideline committee to establish the final 2005 version submitted to Scoliosis, the official Journal of the society, in December 2005.

## Definition

Scoliosis is defined as a lateral curvature of the spine with torsion of the spine and chest as well as a disturbance of the sagittal profile [2].

## Etiology

Idiopathic scoliosis is the most common of all forms of lateral deviation of the spine. By definition, it is a lateral curvature of the spine in an otherwise healthy child, for which a currently recognizable cause has not been found. Less common but better defined etiologies of the disorder include scoliosis of neuromuscular origin, congenital scoliosis, scoliosis in neurofibromatosis, and mesenchymal disorders like Marfan's syndrome [3].

## Epidemiology

The prevalence of adolescent idiopathic scoliosis (AIS), when defined as a curvature greater than 10° according to Cobb, is 2–3%. The prevalence of curvatures greater than 20° is between 0.3 and 0.5%, while curvatures greater than 40° Cobb are found in less than 0.1% of the population. All etiologies of scoliosis other than AIS are encountered more rarely [4].

## Classifications

The anatomical level of the deformity has received attention from clinicians as a basis for scoliosis classification. The level of the apex vertebra (i.e., thoracic, thoracolumbar, lumbar or double major) forms a simple basis for description. In 1983, King and colleagues [5] classified different curvature patterns by the extent of spinal fusion required; however, recent reports have suggested that these classifications lack reliability. Recently, a new description has been developed by Lenke and colleagues [6]. This approach calls for clinical assessment of scoliosis and kyphosis with respect to sagittal profile and curvature components. Systems designed for conservative management include the classifications by Lehnert-Schroth [7] (functional three-curve and functional four-curve scoliosis) and by Rigo [8] (brace construction and application).

## Aims of conservative management

The primary aim of scoliosis management is to stop curvature progression [9]. Improvement of pulmonary function (vital capacity) and treatment of pain are also of major importance. The first of three modes of conservative scoliosis management is based on physical therapy, including Méthode Lyonaise [10], Side-Shift [11], Dobosiewicz [12], Schroth and others [7]. Although discussed from contrasting viewpoints in the international literature, there is some evidence for the effectiveness of scoliosis treatment by physical therapy alone [13].

It has to be emphasized that (1) physical therapy for scoliosis is not just general exercises but rather one of the cited methods designed to address the particular nuances of spinal deformity, and (2) application of such methods requires therapists and clinicians specifically trained and certified in those scoliosis specific conservative intervention methods.

The second mode of conservative management is scoliosis intensive rehabilitation (SIR), which appears to be effective with respect to many signs and symptoms of scoliosis and with respect to impeding curvature progression [14]. The third mode of conservative management is brace treatment, which has been found to be effective in preventing curvature progression and thus in altering the natural history of IS [15,16]. It appears that brace treatment may reduce the prevalence of surgery [17], restore the sagittal profile [18] and influence vertebral rotation [19]. There are also indications that the end result of brace treatment can be predicted [20].

## Systematic application of the modes of conservative treatment with respect to Cobb angle and maturity

**Figure 1:**
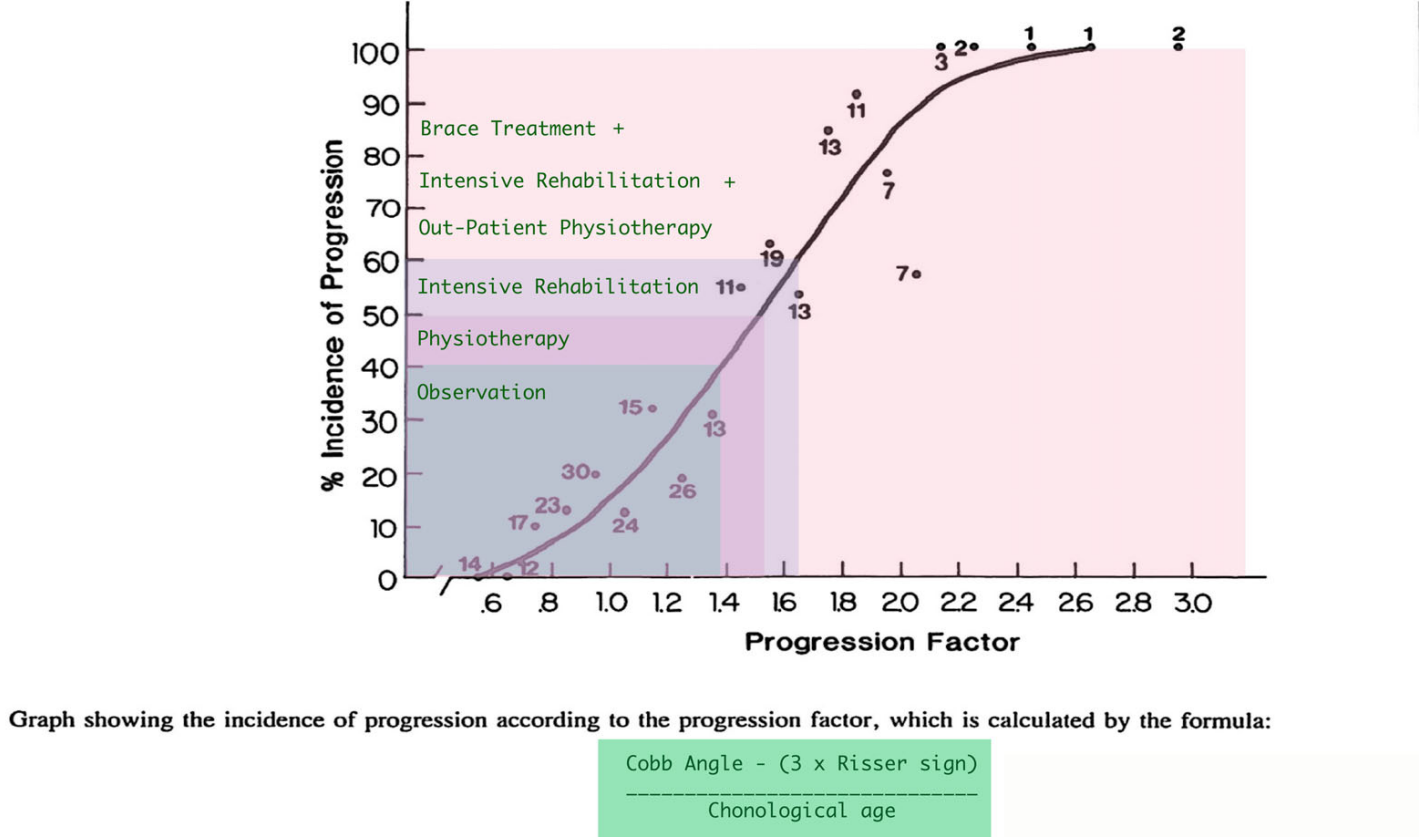
The estimation of the prognostic risk to be used during pubertal growth spurt (modified from Lonstein and Carlson [33]). The numbers in the figure indicate the number of cases that each data point is based on. Note the small number of cases on which the upper margins of the graph are based. Lonstein and Carlson's progression estimation formula is based on curves between 20 and 29 degrees.

Guidelines for conservative intervention are based on current information regarding the risk for significant curvature progression in a given period of time. Each case has its own natural history and must be considered on an individual basis, in the context of a thorough clinical evaluation and patient history [21]. Estimation of risk for progression is based on small (n < 1000) epidemiological surveys in which children were diagnosed with scoliosis, and radiographed periodically to quantify changes in curvature magnitude over time [22-44]. Such surveys support the premise that, among populations of children with a diagnosis of idiopathic scoliosis, risk for progression is highly correlated with potential for growth over the period of observation. In boys, prognosis for progression is more favorable, with relatively fewer individuals having curves that progress to >40 degrees. For SOSORT guidelines, prognostic risk estimation is based on the calculation of Lonstein and Carlson [33]. This calculation is based on curvature progression observed among 727 patients (575 female, 152 male) diagnosed between 1974–1979 in state of Minnesota (United States) school screening programs, and followed until they reached skeletal maturity. (See Figure 1).

### I. Children (no signs of maturity) [21]

a. < 15° Cobb: Observation (6 – 12 month intervals)

b. Cobb angle 15–20°: Outpatient physical therapy with treatment-free intervals (6–12 weeks without physical therapy for those patients at that time have low risk for curve progression). In this context, 'Outpatient physical therapy' is defined here as exercise sessions initiated at the physical therapist's office, plus a home exercise program (two to seven sessions per week according to the physical therapy method being applied). After three months, one exercise session every two weeks may be sufficient.

c. Cobb angle 20–25°: Out patient physiotherapy, scoliosis intensive rehabilitation program (SIR) where available.). SIR, currently available at clinics in Germany and Spain, includes a 3- to 5- week intensive program (4 – 6 hour training sessions per day) for patients with poor prognosis (brace indication, adult with Cobb angle of > 40°, presence of chronic pain).

d. > 25° Cobb: Outpatient physical therapy, scoliosis intensive rehabilitation program (SIR) where available and brace wear (part-time, 12–16 hours)

### II. Children and adolescents, Risser 0–3, first signs of maturation, less than 98% of mature height

The following section is based on progression risk rather than on Cobb angle measurement because of the changing risk profiles for deformityas theskeleton matures. For our purposes, progression risk is calculated by the formula shown in figure 1.

a. Progression risk less than 40%: Observation (3-month intervals)

b. Progression risk 40%: Out patient physiotherapy

c. Progression risk 50%: Out patient physiotherapy, scoliosis intensive rehabilitation program (SIR) where available

d. Progression risk 60%: Out patient physiotherapy, scoliosis intensive rehabilitation program (SIR) where available + part-time brace indication (16 – 23 hours [low risk]).

e. Progression risk 80%: Out patient physiotherapy, scoliosis intensive rehabilitation program (SIR) where available + full-time brace indication (23 hours [high risk])

### III. Children and adolescents presenting with Risser 4 (more than 98% of mature height)

a. < 20° according to Cobb: Observation (6 – 12 Months intervals)

b. 20 – 25° according to Cobb: Outpatient physical therapy

c. > 25° according to Cobb: Outpatient physical therapy, scoliosis intensive rehabilitation programme (SIR) where available

d. > 35° according to Cobb: Outpatient physical therapy, scoliosis intensive rehabilitation programme (SIR) where available + brace (part time, about 16 hours are sufficient)

e. For brace weaning: Outpatient physical therapy, scoliosis intensive rehabilitation programme (SIR) where available + brace with reduced wearing time.

### IV. First presentation with Risser 4–5 (more than 99.5% of mature height before growth is completed)

a. > 25° Cobb: Outpatient physical therapy

b. > 30° Cobb: Outpatient physical therapy, scoliosis intensive rehabilitation program (SIR) where available.

### V. Adults with Cobb angles > 30°

Outpatient physical therapy, scoliosis intensive rehabilitation program (SIR), where available

### VI. Adolescents and adults with scoliosis (of any degree) and chronic pain

Outpatient physical therapy, scoliosis intensive rehabilitation program (SIR) where available, with a special pain program (multimodal pain concept/behavioral + physical concept), brace treatment when a positive effect has been proven [45].

The prognostic estimation and corresponding indications for treatment apply to the most prevalent condition, idiopathic scoliosis. In other types of scoliosis a similar procedure can be applied. Exceptions include those cases where the prognosis is clearly worse, for example in neuromuscular scolioses where a wheelchair is necessary (early surgery for maintaining sitting capability may be required). Other reasons for the consideration of alternative treatments include:

- *Severe decompensation*

- *Severe sagittal deviations with structural lumbar kyphosis ('flatback')*

- *Lumbar, thoracolumbar and caudal component of double curvatures with a disproportionate rotation compared to the Cobb angle and with high risk for future instability at the caudal junctional zone*

- *Severe contractures and muscles shortening*

- *Reduced mobility of the spine especially in the sagittal plane*

- *others to be individually considered *[46]

## Authors' contributions

^*^These authors contributed by reviewing, text editing and adding certain textfiles and references
